# INDUCE-3: A Randomized Phase II/III Study of First-line Feladilimab plus Pembrolizumab in Patients with Recurrent/Metastatic Head and Neck Squamous Cell Carcinoma

**DOI:** 10.1158/1078-0432.CCR-25-1197

**Published:** 2025-12-22

**Authors:** Danny Rischin, Aaron R. Hansen, Ezra E.W. Cohen, Makoto Tahara, Kevin J. Harrington, Robert I. Haddad, Lisa Licitra, Hisham Mehanna, Robert L. Ferris, Piotr Koralewski, Konstantin Penkov, Michael Schenker, David R. Spigel, Amaury Daste, Min Hee Hong, Ye Guo, Michael J. Chisamore, Michael L. Washburn, Amy Phillips‐Jones, John Russell, Sumita Roy‐Ghanta, Catherine Ellis, Marc Ballas, Christophe Le Tourneau

**Affiliations:** 1 Peter MacCallum Cancer Centre and the University of Melbourne, Melbourne, Australia.; 2 Princess Margaret Cancer Centre, Toronto, Canada.; 3 Princess Alexandra Hospital, Brisbane, Australia.; 4 Moores Cancer Center at University of California San Diego, La Jolla, California.; 5 National Cancer Centre Hospital East, Kashiwa, Japan.; 6 The Institute of Cancer Research/The Royal Marsden National Institute for Health and Care Research Biomedical Research Centre, London, United Kingdom.; 7 Dana Farber Cancer Institute, Boston, Massachusett.; 8 Fondazione IRCCS Istituto Nazionale dei Tumori Milan, and University of Milan, Milan, Italy.; 9 Institute of Head and Neck Studies and Education, University of Birmingham, Birmingham, United Kingdom.; 10 UPMC Hillman Cancer Center, Pittsburgh, Pennsylvania.; 11 Wojewodzki Szpital Specjalistyczny im. Ludwika Rydygiera w Krakowie, Krakow, Poland.; 12 Private Medical Institution Euromedservice, St. Petersburg, Russia.; 13 Centrul de Oncologie Sf. Nectarie, Oncologie Medicala, Craiova, Romania.; 14 University of Medicine and Pharmacy of Craiova, Craiova, Romania.; 15 Sarah Cannon Research Institute, Nashville, Tennessee.; 16 Hôpital Saint-André, University of Bordeaux-CHU de Bordeaux, Bordeaux, France.; 17 Yonsai Cancer Center, Severance Hospital, Yonsei University Health System, Seoul, Korea.; 18 Shanghai East Hospital, School of Medicine, Tongji University, Shanghai, China.; 19 Merck & Co., Inc., Rahway, New Jersey.; 20 GSK, Collegeville, Pennsylvania.; 21 GSK, Stevenage, United Kingdom.; 22 Department of Drug Development and Innovation (D3i), Institut Curie, Paris-Saclay University, Paris, France.

## Abstract

**Purpose:**

Feladilimab, an inducible T-cell costimulatory receptor agonist, demonstrated clinical activity in combination with pembrolizumab in a phase I head and neck squamous cell carcinoma (HNSCC) expansion cohort, prompting further evaluation in patients in this setting.

**Patients and Methods:**

INDUCE-3 (NCT04128696) was a randomized, double-blind, phase II/III study in patients with first-line PD-L1–positive recurrent and/or metastatic HNSCC. A 2-in-1 adaptive design was implemented, with an option to expand the phase II study into a phase III confirmatory study. Patients were randomized 1:1 to receive feladilimab plus pembrolizumab or placebo plus pembrolizumab, with up to 35 cycles of treatment for approximately 2 years. Primary endpoints were overall survival (OS) and investigator-assessed progression-free survival (PFS).

**Results:**

The study enrolled 315 patients. Following the review of unblinded interim data in 140 patients, an Independent Data Monitoring Committee recommended stopping patient accrual based on prespecified criteria. Existing patients discontinued feladilimab or placebo. Pembrolizumab treatment continued until prespecified stopping criteria were met. This study demonstrated no evidence of an effect in favor of feladilimab plus pembrolizumab, with an adjusted HR of 1.51 for OS and 1.40 for PFS (median OS: 44.1 weeks [95% confidence interval (CI), 35.9–not applicable]; median PFS: 10.1 weeks [95% CI, 9.1–15]) versus placebo plus pembrolizumab [median OS: not reached; median PFS: 16 weeks (95% CI, 14.3–26.1)]. The incidence of treatment-related adverse events was higher in the placebo group.

**Conclusions:**

This analysis does not support the combination of feladilimab plus pembrolizumab due to a lack of superiority over placebo plus pembrolizumab.

**Translational Relevance**

Pembrolizumab, a programmed cell death protein 1 (PD-1) inhibitor, with or without chemotherapy, improved survival outcomes as first-line (1L) treatment for recurrent/metastatic (R/M) head and neck squamous cell carcinoma (HNSCC) versus standard of care. Due to inherent or emerging resistance, there remains a need for 1L immunotherapies. Combining immunotherapies targeting different components of the tumor immunity cycle could help overcome immune suppression. Feladilimab, an inducible T-cell costimulatory receptor (ICOS) agonist, demonstrated clinical activity in combination with pembrolizumab in a phase I HNSCC expansion cohort. INDUCE-3 evaluated whether the addition of 1L feladilimab to pembrolizumab improved pembrolizumab efficacy in patients with PD-L1–positive (R/M) HNSCC. Following the review of unblinded interim data, an Independent Data Monitoring Committee recommended stopping patient accrual based on prespecified criteria. There was no evidence of a favorable treatment response to combined feladilimab plus pembrolizumab across primary or key secondary endpoints. These results raise doubts about ICOS as a target in HNSCC.

## Introduction

The programmed cell death protein 1 (PD-1) inhibitor pembrolizumab, with or without chemotherapy, has improved overall survival (OS) for patients receiving first-line (1L) treatment for recurrent/metastatic (R/M) head and neck squamous cell carcinoma (HNSCC) compared with the previous standard of care (SOC; ref. 1). This new SOC has been adopted globally; however, a significant need remains for new 1L treatments owing to inherent or emerging resistance to immune checkpoint blockade, with only a minority of patients deriving durable benefit (2).

Combining immunomodulatory agents targeting different components of the tumor immunity cycle could help overcome immune suppression, preventing an effective antitumor response (3). This approach has been successful in melanoma treatment with nivolumab in combination with ipilimumab or relatlimab, but the ipilimumab/nivolumab combination was unsuccessful in HNSCC (4–6).

Inducible T-cell costimulatory receptor (ICOS) is a costimulatory receptor promoting T-cell proliferation and survival, making it a promising target for immunotherapy (7, 8). The ICOS ligand binds to ICOS with high affinity, and its expression is largely restricted to antigen-presenting cells (9). PD-1 and ICOS are overexpressed in HNSCC, and OS rates are higher in patients with high ICOS-expressing tumors compared with those with low expression (10). Feladilimab (GSK3359609), a humanized ICOS agonist IgG4 mAb (11), demonstrated antitumor activity in combination with PD-1 inhibition in nonclinical models (12). In the HNSCC expansion cohort of INDUCE-1 (NCT02723955), feladilimab monotherapy demonstrated a manageable safety profile in patients with PD-1/programmed cell death ligand 1 (PD-L1)–experienced HNSCC and a promising overall response rate (ORR) in combination with pembrolizumab (28%), providing the clinical rationale for INDUCE-3 (NCT04128696; ref. 13).

INDUCE-3 evaluated whether the addition of feladilimab to pembrolizumab as 1L treatment improved pembrolizumab efficacy in patients with PD-L1–positive R/M HNSCC.

## Patients and Methods

### Study design

INDUCE-3 was a randomized, double-blind, phase II/III study in patients with PD-L1+ R/M HNSCC conducted at 135 sites across 25 countries. The 2-in-1 adaptive design (14) allowed expansion from phase II into a phase III confirmatory study without changing eligibility criteria, endpoints, or randomization. Following screening, patients were stratified by PD-L1 combined positive score (CPS; CPS ≥ 20 vs. 1 ≤ CPS < 20) and human papillomavirus (HPV) status for oropharyngeal cancers. PD-L1 CPS status was quantified using the 22C3 pharmDx immunohistochemistry (IHC) assay by central laboratory testing, and HPV status for oropharyngeal cancers (positive or negative/unknown) was assessed via the p16 IHC assay. Eligible patients were randomized 1:1 to feladilimab plus pembrolizumab (feladilimab–pembrolizumab) or placebo plus pembrolizumab (placebo–pembrolizumab) to receive up to 35 treatment cycles for approximately 2 years. Randomization occurred centrally using an interactive response technology. Patients and investigators were blinded to the assigned study treatment for the duration of the study. Follow-up (minimum 6 months) was initiated once study treatment was permanently discontinued.

This study was approved by a national, regional, or investigational center ethics committee or Institutional Review Board and conducted in accordance with the International Council for Harmonisation of Technical Requirements for Pharmaceuticals for Human Use Good Clinical Practice, the Declaration of Helsinki, and applicable country-specific regulatory requirements, including US 21 Code of Federal Regulations 312.3(b) for the constitution of independent ethics committees. All patients provided written informed consent before enrollment.

### Patients

Eligible patients were ≥18 years old; had pathologically confirmed R/M squamous cell carcinoma of the oropharynx, oral cavity, hypopharynx, or larynx that was not curable by local therapy; were suitable for 1L treatment for R/M HNSCC; had measurable disease per Response Evaluation Criteria in Solid Tumors (RECIST) version 1.1; had a life expectancy ≥12 weeks; had an Eastern Cooperative Oncology Group Performance Status (ECOG PS) score of 0 to 1; had a PD-L1 CPS ≥1; and had known HPV status for oropharyngeal cancer.

Patients were excluded if they had received prior systemic treatment for R/M HNSCC, except for those patients who completed treatment >6 months prior, if given as part of multimodal treatment for locally advanced disease, and had no disease progression or recurrence within 6 months of systemic treatment completion. After trial commencement, the protocol was amended (May 19, 2020) to include eligibility criteria to restrict the population to those with recurrence >6 months from completion of systemic therapy to address complications of rapid disease progression. Additional exclusion criteria included grade 3 or 4 hypercalcemia; major surgery within 28 days prior to randomization; invasive malignancy or history of invasive malignancy other than the disease under study within the past 3 years; toxicity from previous anticancer treatment that included grade 3 or 4 toxicity considered related to prior immunotherapy and that led to treatment discontinuation; toxicity related to prior treatment that has not resolved to ≤grade 1; and/or any serious (grade ≥ 3) and/or unstable preexisting medical condition other than from malignancy. Patients at high risk of tumor hemorrhagic events were excluded.

### Treatments

Upon randomization, patients received feladilimab 24 mg plus pembrolizumab 200 mg or placebo plus pembrolizumab 200 mg as an intravenous infusion over 30 minutes once every 3 weeks. Feladilimab or placebo was administered first, followed by pembrolizumab. Disease assessments were conducted once every 6 weeks between weeks 9 and 51 and once every 12 weeks thereafter. Patient-reported outcomes (PRO) were assessed on day 1, once every 3 weeks up to week 21, and once every 6 weeks thereafter. Patients with disease progression according to RECIST version 1.1 were permitted to continue study treatment for up to 35 cycles or until progression by immune-based RECIST guidelines, subject to investigator discretion and patient clinical stability.

### Endpoints

Primary endpoints were OS (time from randomization to death due to any cause) in the modified intention-to-treat (mITT) population (all patients who received study intervention excluding those first dosed after the date of discontinuation) and CPS ≥ 20 populations and investigator-assessed progression-free survival (PFS; time from randomization to first documented disease progression or death due to any cause) in the mITT population.

Secondary endpoints included investigator-assessed PFS (per RECIST version 1.1 in CPS ≥ 20); investigator-determined ORR [partial response (PR) or complete response (CR)]; investigator-assessed disease control rate (DCR; stable disease for ≥15 weeks, PR, or CR); health-related quality of life (HRQoL) assessments; and safety and tolerability. HRQoL assessments included time to deterioration (TTD) in pain measured by the European Organisation for Research and Treatment of Cancer (EORTC) quality of life questionnaire (QLQ)-H&N35 pain (a head and neck cancer–specific module with multi-item scales) in the mITT population and CPS ≥ 20 subgroup and TTD in physical function measured by the PRO Measurement Information System (PROMIS) Physical Function 8c (PF8c) score. As no threshold for meaningful within-individual change is established for the EORTC Item Library (IL) 51 (a subset of domains from the EORTC QLQ-H&N35) pain domain score or PROMIS-PF8c score, the value for use in the TTD analyses was determined using pooled blinded data from INDUCE-3 (GSK Study 209229) and INDUCE-4 (GSK Study 209227). For the EORTC IL51 pain domain, a meaningful change threshold of 8.33 was selected based on recommended anchor- and distribution-based methods (unpublished data). This corresponds with one observable change (e.g., one category) on any item in the domain. A definitive, meaningful deterioration was defined as an increase from baseline at the defined threshold that was observed at all subsequent nonmissing visits. A meaningful change threshold of 2.4 for TTD in physical functioning was selected based on recommended anchor- and distribution-based methods (unpublished data). This threshold corresponds with two observable changes on the measure. A definitive, meaningful deterioration is defined as a decrease from baseline at the defined threshold that is observed at all subsequent nonmissing visits.

Safety endpoints included adverse events (AEs).

Exploratory endpoints included blood and tumor biomarker analyses (in those patients providing archival tumor tissue) with circulating tumor DNA (ctDNA) tumor mutational burden (TMB) analysis at baseline using the OMNI assay and changes with molecular response (≥50% reduction from baseline at week 15 in ctDNA using the REVEAL assay; refs. 15,16). Tumor sequencing was performed using the ImmunoID NeXT platform.

#### Germline biomarker analysis (PGx)

Genomic DNA was extracted from the peripheral blood of participants consenting to genetic research using the Gentra Puregene Kits (QIAGEN) by Q2 solution. The Affymetrix Axiom Precision Medicine Research Array was used for genotyping by Azenta Life Sciences, formerly Brooks Life Science. The human leukocyte antigen (*HLA*) alleles were imputed by using the array data and the HIBAG algorithm (17) with a reference set of haplotypes. *HLA-I* heterozygosity status was determined based on the imputed alleles in *HLA-A*, *HLA-B*, and *HLA-C*, coded as binary (i.e., “Het” if heterozygous at all three gene loci vs. “Hom” if homozygous at least in one gene locus). Similarly, the *HLA* evolutionary divergence mean score was calculated based on imputed *HLA-I* alleles using the method described in Pierini and Lenz (18) and implemented in Chowell and colleagues (19). Polygenic risk scores (PRS) were calculated for each participant as the weighted sum of the number of high-risk alleles at selected loci. PRS for psoriasis, vitiligo, and atopic dermatitis were calculated based on the methodology outlined in Khan and colleagues (20). Similarly, the PRS for hypothyroidism was calculated based on the methodology and genetic instruments described in Khan and colleagues (21).

All genotype calling and quality control were performed in accordance with the manufacturers’ protocols. All the vendors involved in experimental data generation did so through a fee-for-service agreement.

#### Analysis subgroups

Analyses subgroups included (i) patients in the mITT population, who received at least one dose of their allocated treatment, provided written informed consent for genetics research and a DNA sample, and have been successfully genotyped; (ii) White-only patients; or (iii) patients on 22 weeks of follow-up from the date of randomization to the time of data cut. Clinical endpoints included PFS, OS, and radiographic response indicating clinical benefit (the best overall response of CR, PR, or stable disease recorded from the date of randomization until disease progression or initiation of new anticancer therapy). Not evaluable (NE) and not applicable (NA) entries were excluded from the analysis. Demographic and baseline variables such as ECOG PS, albumin high/low category, alkaline phosphatase high/low category, neutrophil-to-lymphocyte ratio, gender, as well as the top 10 genome-wide association study genetic principal components to account for potential ancestry differences in the study population were evaluated and included in the regression model. Differences in biomarker distributions between study arms were assessed using linear models and Fisher’s exact tests for continuous and discrete variables, respectively. Cox regression, adjusting for baseline variables shown to significantly impact survival outcomes, was used to test for association. Bidirectional stepwise regression was used to select significant covariates to be included in the Cox model for each survival outcome. Representative data for *HLA* biomarkers in the mITT population for patients treated with pembrolizumab/control are presented in this article (Supplementary Fig. S1).

#### Prespecified protocol defined statistical adaptive decision criteria

The analysis for adaptive design was conducted using ORR/DCR when approximately the first 100 participants had a minimum follow-up of 6 months.

The adaptive decision criteria were positive if there was at least an 8% improvement in ORR in the feladilimab plus pembrolizumab group compared with the pembrolizumab plus placebo in the mITT population. Confirmation of CR and PR was not required in the adaptive decision-making.

1. If the ORR outcome per RECIST version 1.1 was positive with ΔORR ≥ 8% in the mITT population, the study will continue to an originally planned phase III sample size for a definitive phase III evaluation.
2. If the ORR/DCR outcome per RECIST version 1.1 was negative with ΔORR < 0% and ΔDCR < 0% in the mITT population, the study may stop for futility depending on the recommendation of the Independent Data Monitoring Committee (IDMC) based on the totality of the data.
3. Otherwise, the study will continue as planned with a phase II sample size for a definitive phase II evaluation.

### Statistical analysis

A sample size of 374 patients was estimated for the phase II study, assuming a prevalence rate of 53% for PD-L1 CPS ≥ 20 among the mITT population. The study was event-driven, and the sample size calculation was driven by OS events. The assumptions for the sample size and power calculation applied to the study, whether phase II or phase III. Endpoints were analyzed using the stratified log-rank test and Cox proportional hazards model with Efron’s tie handling method to assess treatment differences. ORR and DCR were assessed in the mITT population using the stratified Miettinen and Nurminen method with strata weighting by sample size. Efficacy data are presented for the mITT population; *P* values are not presented due to no hypothesis testing planned at this interim analysis. Safety was assessed in the safety population (all patients who received at least one dose of allocated study treatment) with descriptive summaries by the treatment group. ctDNA analyses were performed *post hoc* in the biomarker-evaluable population (BEP) 1 (patients with a baseline plasma sample) and BEP2 (patients with both a baseline and week 16 plasma sample).

## Results

### Patients

**Figure 1.**
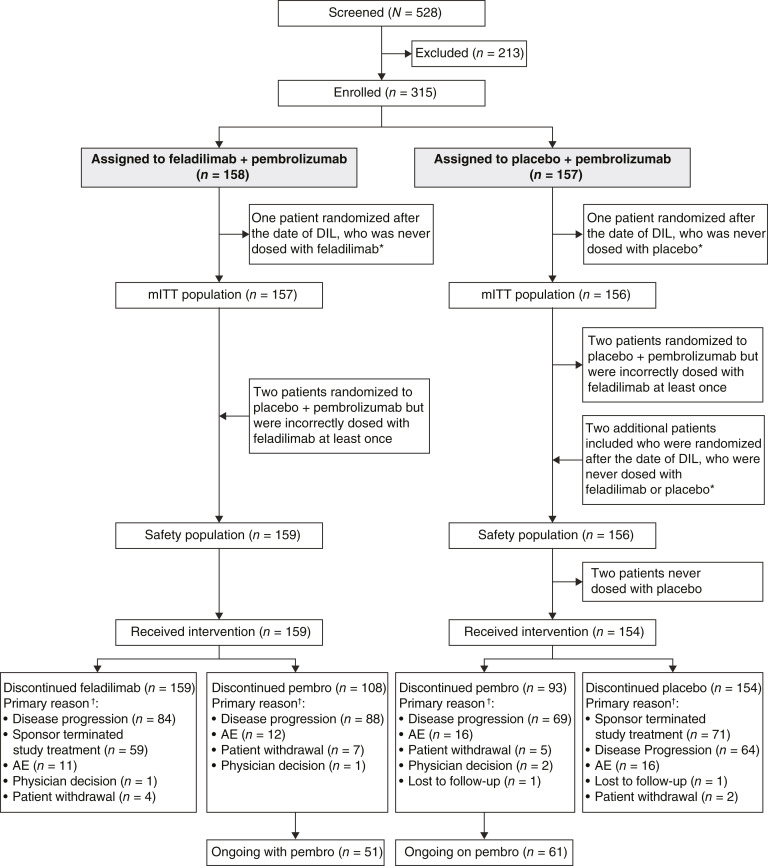
Study design. *Patients randomized after the date of DIL who were unable to receive feladilimab/placebo were excluded from the mITT population but included in the safety population; ^†^more than one reason could be selected; AE, adverse event; DIL, Dear Investigator Letter; ITT, intention to treat; mITT, modified ITT.

The first patient was enrolled on November 21, 2019, and at data cutoff (April 27, 2021), 315 patients were enrolled (mITT population, *n* = 313; safety population, *n* = 315; Fig. 1). In the mITT population, 89 patients (28%) completed the study, 14 patients (4%) withdrew, and 210 patients (67%) remained in the study. The most common reason for withdrawal was patient decision (4%).

On April 12, 2021, the IDMC recommended stopping accrual following a safety and efficacy data review from an unblinded interim analysis of 140 patients who had completed a minimum of 22 weeks of follow-up. At this time, the study met prespecified, protocol-defined statistical futility. On April 13, 2021, investigators were instructed to stop screening and randomizing further patients to this study and to cease feladilimab dosing. Patients enrolled could continue pembrolizumab until disease progression, death, or unacceptable toxicity.

Patients received a median of 5 (range: 1–19) and 6 (range: 1–23) treatment cycles in the feladilimab–pembrolizumab and placebo–pembrolizumab groups, respectively.

### Baseline demographics

Baseline characteristics are presented for the mITT population (Table 1). Demographic characteristics were generally balanced between treatment groups and were representative of the general HNSCC population (Supplementary Table S1). The most common primary tumor types were oropharynx (38%) and lip/oral cavity cancer (30%). The disease recurrence patterns were local/regional recurrence (24%), metastatic (29%), both (25%), and *de novo* metastatic (22%).

**Table 1.** Baseline demographics and disease characteristics (mITT population).

Characteristics, *n* (%) unless otherwise specified	Feladilimab plus pembrolizumab (*n* = 157)	Placebo plus pembrolizumab (*n* = 156)	Total (*n* = 313)
Age, years^a^, median (range)	62 (25–86)	64 (45–88)	63 (25–88)
Sex			
Female	29 (18)	31 (20)	60 (19)
Male	128 (82)	125 (80)	253 (81)
Race			
White	116 (74)	120 (77)	236 (75)
Asian	33 (21)	28 (18)	61 (19)
Black or African American	4 (3)	2 (1)	6 (2)
Mixed race	1 (<1)	0	1 (<1)
Missing	3 (2)	6 (4)	9 (3)
Region of enrollment^b^			
North America	31 (20)	27 (17)	58 (19)
Europe	74 (47)	91 (58)	165 (53)
Asia	31 (20)	28 (18)	59 (19)
Rest of the world	21 (13)	10 (6)	31 (10)
Smoking status			
Never smoked	33 (21)	25 (16)	58 (19)
Current smoker	30 (19)	33 (21)	63 (20)
Former smoker	93 (59)	98 (63)	191 (61)
Missing	1 (<1)	0	1 (<1)
ECOG PS			
0	58 (37)	52 (33)	110 (35)
1	99 (63)	104 (67)	203 (65)
Primary tumor type^c^			
Oropharynx	59 (38)	60 (38)	119 (38)
Non-oropharynx	98 (62)	96 (62)	194 (62)
PD-L1 CPS status^c^			
1 ≤ CPS < 20	89 (57)	86 (56)	175 (57)
CPS ≥ 20	67 (43)	67 (44)	134 (43)
Prior therapy			
Radiotherapy	112 (71)	109 (70)	221 (71)
Chemotherapy	75 (48)	76 (49)	151 (48)
Primary tumor type under study			
Laryngeal cancer	32 (20)	29 (19)	61 (19)
Lip and/or oral cavity cancer	46 (29)	48 (31)	94 (30)
Hypopharyngeal cancer	20 (13)	19 (12)	39 (12)
Oropharynx cancer^d^	59 (38)	60 (38)	119 (38)
HPV-positive oropharynx cancer	35 (59)	33 (55)	68 (57)
HPV-negative oropharynx cancer	24 (41)	26 (43)	50 (42)
Disease recurrence type			
Locally/regional recurrent	38 (24)	38 (24)	76 (24)
Metastatic	52 (33)	38 (24)	90 (29)
Both locally/regional recurrent and metastatic	30 (19)	47 (30)	77 (25)
No (*de novo* metastatic)	37 (24)	33 (21)	70 (22)
Time since last recurrence, days, median (range)^e^	51.0 (19–975)	53.5 (5–2,250)	52.0 (5–2,250)

a Age was imputed when the full date of birth is not provided.

b America: USA and Canada; Europe: Greece, Germany, Spain, France, UK, Italy, Denmark, Ireland, Poland, Romania, Portugal, Netherlands, Norway, and Switzerland; rest of world: Mexico, Taiwan, China, South Korea, Japan, Israel, Russia, Australia, Brazil, and Argentina.

c Source data for HPV status (based on p16 IHC) and CPS status (based on PD-L1 IHC) from laboratory data and from case report forms for primary tumor location.

d HPV status was unconfirmed in one patient in the placebo plus pembrolizumab group.

e Defined as the randomization date minus the last recurrence date plus 1.

### Efficacy

**Figure 2.**
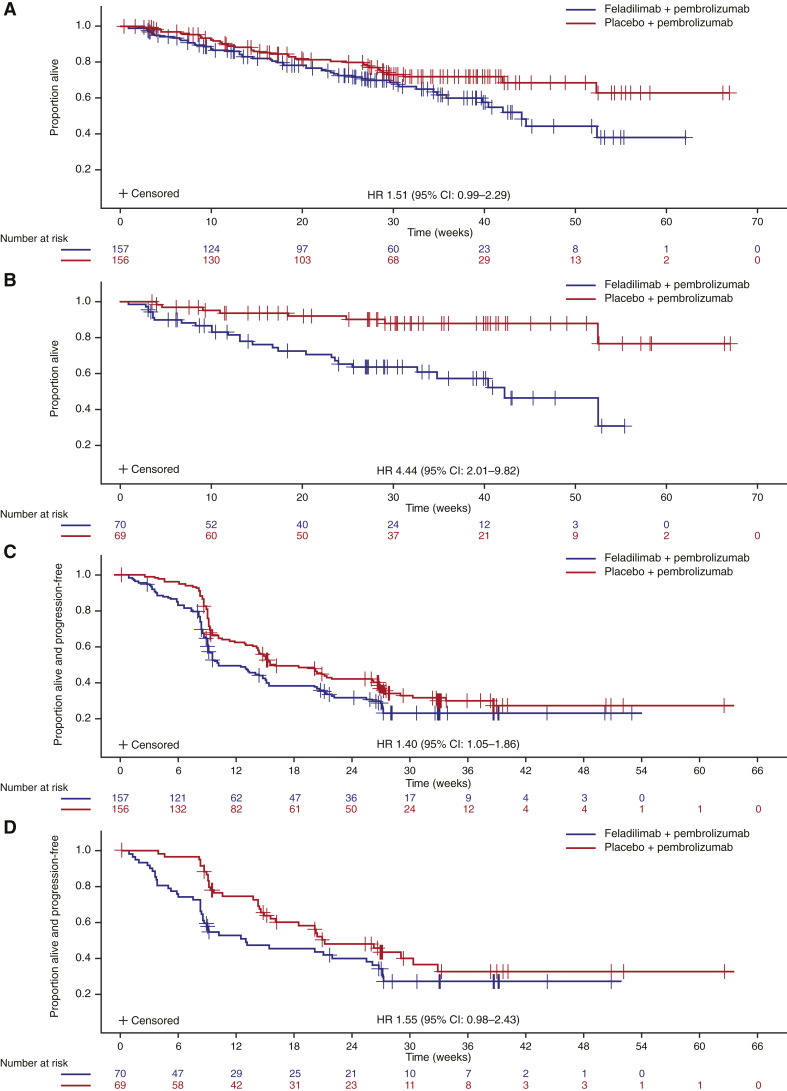
OS and PFS for the mITT population (**A** and **C**) and the PD-L1 CPS ≥ 20 subgroup (**B** and **D**). Data cutoff: April 27, 2021. The mITT population included all randomized patients who received the study intervention, whether or not randomized, but excluded those who were first dosed or randomized after the date of requesting immediate discontinuation of feladilimab and placebo. CI, confidence interval; HR, hazard ratio; mITT, modified intention-to-treat; OS, overall survival; PD-L1 CPS, programmed cell death ligand-1 combined positive score; PFS, progression-free survival; RECIST v1.1, Response Evaluation Criteria in Solid Tumors version 1.1.

At data cutoff, the median duration of follow-up was 6.28 months (range: 0.1–15.4). In the mITT population, the estimated median OS in the feladilimab–pembrolizumab group was 44.1 weeks [95% confidence interval (CI), 35.9–NA]. A higher proportion of patients in the feladilimab–pembrolizumab group experienced an event (*n* = 54, 34%) versus those receiving placebo–pembrolizumab (*n* = 38, 24%). In the PD-L1 CPS ≥ 20 subgroup, the estimated median OS in the feladilimab–pembrolizumab group was 42.1 weeks (95% CI, 25.4–NA), with 27 (39%) patients experiencing an event. The estimated median OS was not reached for the placebo–pembrolizumab group, with eight (12%) patients experiencing an event. Adjusted hazard ratios (HR) for the feladilimab–pembrolizumab and placebo–pembrolizumab arms were 1.51 (95% CI, 0.99–2.29) in the mITT population and 4.44 (95% CI, 2.01–9.82) in the PD-L1 CPS ≥ 20 subgroup (Fig. 2A and B).

At data cutoff, the estimated median PFS in the feladilimab–pembrolizumab group was 10.1 weeks (95% CI, 9.1–15.0) compared with 16.0 weeks (95% CI, 14.3–26.1) in the placebo–pembrolizumab group, with an adjusted HR of 1.40 (95% CI, 1.05–1.86) for the mITT population (Fig. 2C). The estimated median PFS in the PD-L1 CPS ≥ 20 subgroup was 13.0 weeks (95% CI, 8.6–26.1) in the feladilimab–pembrolizumab group compared with 21.1 weeks (95% CI, 15.6–32.9) in the placebo–pembrolizumab group, with an adjusted HR of 1.55 (95% CI, 0.98–2.43; Fig. 2D).

In the mITT population, ORR was 19.7% (95% CI, 13.8–26.8) in the feladilimab–pembrolizumab group compared with 25.0% (95% CI, 18.4–32.6) in the placebo–pembrolizumab group (Table 2). The estimated difference in ORR between treatment groups was −5.3% (95% CI, −14.6 to 4). In the PD-L1 CPS ≥ 20 subgroup, the ORR was 20% (95% CI, 11.4–31.3) in the feladilimab–pembrolizumab group compared with 33.3% (95% CI, 22.4–45.7) in the placebo–pembrolizumab group. The estimated difference in ORR between treatment groups was −13.3% (95% CI, −27.8 to 1.5).

**Table 2.** Summary of investigator-assessed best response without confirmation (mITT population and PD-L1 CPS ≥ 20 subgroup).

Response	mITT population		PD-L1 CPS ≥ 20 subgroup	
	Feladilimab plus pembrolizumab (*n* = 157)	Placebo plus pembrolizumab (*n* = 156)	Feladilimab plus pembrolizumab (*n* = 70)	Placebo plus pembrolizumab (*n* = 69)
Best response, *n* (%)				
CR	3 (2)	4 (3)	3 (4)	2 (3)
PR	28 (18)	35 (22)	11 (16)	21 (30)
SD	40 (25)	48 (31)	16 (23)	24 (35)
SD ≥ 15 weeks^a^	22 (14)	32 (21)	12 (17)	15 (22)
PD	57 (36)	41 (26)	24 (34)	12 (17)
NE^b^	29 (18)	28 (18)	16 (23)	10 (14)
ORR^c^, *n* (%; 95% CI)	31 (19.7; 13.8 to 26.8)	39 (25; 18.4 to 32.6)	14 (20; 11.4 to 31.3)	23 (33.3; 22.4 to 45.7)
Difference in ORR (95% CI)^d^	−5.3% (−14.6 to 4)	—	−13.3% (−27.8 to 1.5)	—
DCR^c^, *n* (%; 95% CI)	52 (33.1; 25.8 to 41.1)	70 (44.9; 36.9 to 53)	26 (37.1; 25.9 to 49.5)	38 (55.1; 42.6 to 67.1)
Difference in DCR (95% CI)^d^	−11.8% (−22.4% to −1.1%)	​	−18.0% (−33.7 to −1.4)	​

Data cutoff: April 27, 2021. The mITT population included all randomized patients who received the study intervention, whether or not randomized, but excluded those who were first dosed or randomized after the date of requesting immediate discontinuation of feladilimab and placebo.

Abbreviations: PD, progressive disease; SD, stable disease.

a A 1-week visit window was considered for the duration of SD (i.e., 14 weeks for SD ≥ 15 weeks).

b Includes patients who were NE (had at least one post-baseline response assessment, and none of the investigator assigned responses qualify to be CR, PR, SD, or PD based on the defined criteria) and those with no assessment (no post-baseline tumor assessment was performed).

c ORR was defined as a PR or CR, and DCR was defined as SD for ≥15 weeks, PR, or CR per RECIST version 1.1. Rate and associated two-sided 95% CIs for each treatment arm are unadjusted. Two-sided 95% exact (Clopper–Pearson) CIs are provided.

d The weighted difference in ORR/DCR and associated two-sided 95% CIs are from the stratified Miettinen and Nurminen method with strata weighting by sample size.

In the mITT population, DCR was 33.1% (95% CI, 25.8–41.1) in the feladilimab–pembrolizumab group compared with 44.9% (95% CI, 36.9–53) in the placebo–pembrolizumab group (Table 2). The estimated difference in DCR was −11.8% (95% CI, −22.4 to −1.1). In the PD-L1 CPS ≥ 20 subgroup, the DCR was 37.1% (95% CI, 25.9–49.5) in the feladilimab–pembrolizumab group compared with 55.1% (95% CI, 42.6–67.1) in the placebo–pembrolizumab group. The estimated difference in DCR was −18.0% (95% CI, −33.7 to −1.4).

### Patient-reported HRQoL outcomes

The estimated median TTD in pain (mITT population) was 6.3 months (95% CI, 5.1–NA) in the feladilimab–pembrolizumab group compared with 10.4 months (95% CI, 6.3–NA) in the placebo–pembrolizumab group, with an adjusted HR of 1.17 (95% CI, 0.78–1.77; Supplementary Table S2).

The estimated median TTD in physical function (mITT population) was comparable (4.9 months) between treatment groups, with an adjusted HR of 0.91 (95% CI, 0.62–1.34; Supplementary Table S2).

### Safety

AE incidence was similar between treatment groups, except for treatment-related (TR) AEs (TRAE) and AEs of special interest (AESI), which were higher in the placebo–pembrolizumab group (57% and 40%, respectively) compared with the feladilimab–pembrolizumab group (48% and 26%, respectively; Table 3). The incidence of AEs occurring in ≥2% of patients per treatment group, AEs by grade, and serious AEs were similar between groups (Table 3; Supplementary Tables S3 and S4).

**Table 3.** Summary of AEs (safety population).

*n* (%)	Feladilimab plus pembrolizumab (*n* = 159)	Placebo plus pembrolizumab (*n* = 156)	Total (*n* = 315)
Any AE	145 (91)	140 (90)	285 (90)
TRAEs	76 (48)	89 (57)	165 (52)
Grade ≥ 3 AEs	60 (38)	62 (40)	122 (39)
TR grade ≥ 3 AEs	17 (11)	18 (12)	35 (11)
AEs leading to permanent discontinuation of study treatment	10 (6)	16 (10)	26 (8)
Treatment-related AEs leading to permanent discontinuation of study treatment	1 (<1)	7 (4)	8 (3)
AEs leading to dose interruption/delay	27 (17)	36 (23)	63 (20)
Any SAE	46 (29)	47 (30)	93 (30)
TR SAEs	12 (8)	13 (8)	25 (8)
Fatal SAEs	13 (8)	15 (10)	28 (9)
TR fatal SAEs	2 (1)	1 (<1)	3 (<1)
Grade ≥ 3 SAEs	39 (25)	38 (24)	77 (24)
Any AESI	41 (26)	63 (40)	104 (33)
Most common AESI (≥2%)^a^	​	​	​
Rash	9 (6)	18 (12)	27 (9)
Hypothyroidism	11 (7)	15 (10)	26 (8)
Pruritus	9 (6)	14 (9)	23 (7)
Arthralgia	10 (6)	10 (6)	20 (6)
Hyperthyroidism	4 (3)	7 (4)	11 (3)
Stomatitis	3 (2)	7 (4)	10 (3)
Hyperglycemia	3 (2)	3 (2)	6 (2)
Pneumonitis	2 (1)	3 (2)	5 (2)
Rash maculopapular	1 (<1)	3 (2)	4 (1)

Data cutoff: April 27, 2021. Safety population includes all patients who received at least one dose of allocated study treatment. Discontinuation of treatment reflects the discontinuation of at least one component of the study treatment administered. Similarly, the relationship to treatment reflects the relationship to at least one component.

Abbreviation: SAE, serious AE.

a Preferred term listed in decreasing frequency in the total group of patients.

The most frequently reported TR AESIs (≥10 patients in either of the treatment groups) were hypothyroidism, pruritus, and rash. Most AESIs were grade 1/2 events. Grade ≥3 AESIs were reported in one patient in the feladilimab–pembrolizumab group (colitis) and three patients in the placebo–pembrolizumab group (acute kidney injury, pneumonitis, and hepatitis).

Two TR deaths occurred in the feladilimab–pembrolizumab group (respiratory failure and small intestinal perforation) and one in the placebo–pembrolizumab group (mouth hemorrhage).

During the study, 92 (29%) patients died, with a higher number of deaths in the feladilimab–pembrolizumab group (34%) than in the placebo–pembrolizumab group (24%). The primary cause of death in both groups was the disease under study, and most occurred ≥30 days after the last dose of study treatment.

### Exploratory endpoints

In total, 145 and 97 patient samples were included in BEP1 and BEP2, respectively. Clinical characteristics in the BEP populations were similar to the mITT population; however, BEP1 had a lower median baseline tumor burden (42.5 vs. 53.5 mm in the feladilimab–pembrolizumab arm; 49 vs. 56 mm in the placebo–pembrolizumab arm). Baseline ctDNA and TMB were balanced between treatment groups (Supplementary Fig. S1).

**Figure 3.**
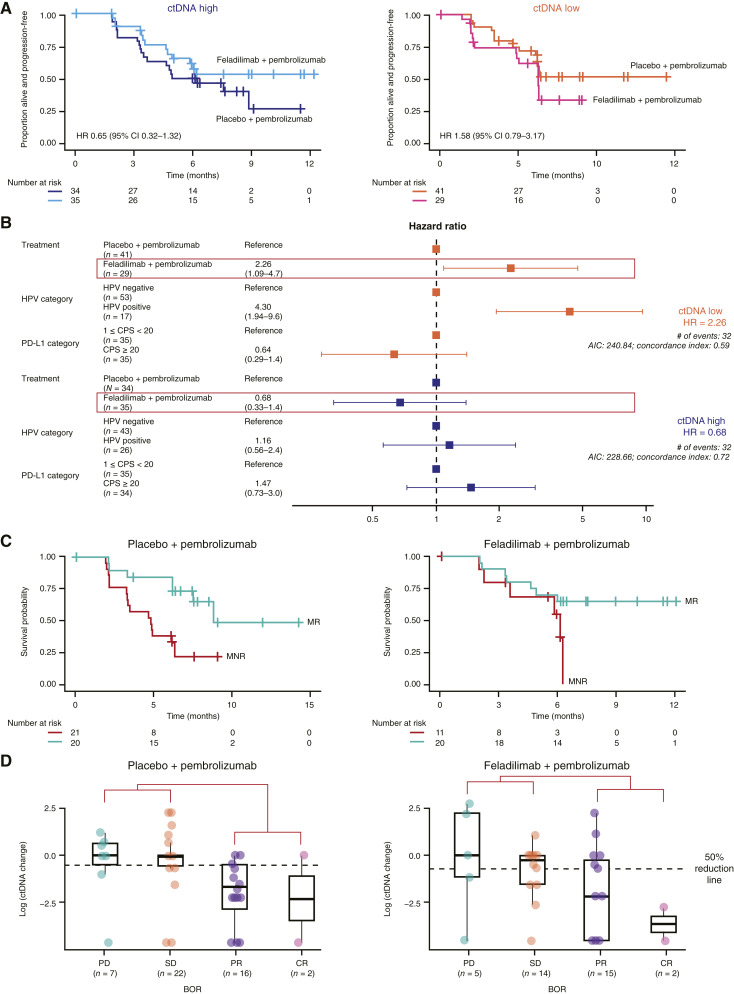
Patient outcomes by baseline ctDNA and molecular response (*post hoc* analyses). **A,** PFS Kaplan–Meier curves for the BEP1 stratified by baseline ctDNA. **B,** PFS treatment responses in patients stratified by baseline ctDNA level, adjusted for PD-L1 and HPV status. **C,** PFS Kaplan–Meier curves for the BEP2 stratified by molecular responder status at week 15. **D,** BOR distribution across treatment groups. BEP1 is defined as all patients with a baseline sample, and the BEP2 is defined as all patients with both a baseline and week 15 sample. ctDNA high and low are defined as mVAF ≥ 1.33% and <1.33%, respectively. Molecular responders and nonresponders are defined as patients with a reduction from baseline at week 15 in ctDNA of ≥50% and <50%, respectively. AIC, Akaike information criterion; BOR, best overall response; MNR, molecular nonresponder; MR, molecular responder; PD, progressive disease; SD, stable disease.

Within the high baseline ctDNA group [mean variant allele frequency (mVAF) > mean mVAF of BEP1 (1.33%)], longer PFS was observed in the feladilimab–pembrolizumab group compared with the placebo–pembrolizumab group [HR: 0.65 (95% CI, 0.32–1.32)], whereas within the low baseline ctDNA group (≤1.33%), the opposite effect was observed, with longer PFS in the placebo–pembrolizumab group compared with the feladilimab–pembrolizumab group [HR: 1.58 (95% CI, 0.79–3.17); Fig. 3A]. These findings held after adjusting for PD-L1 and HPV status, with longer PFS observed in the placebo–pembrolizumab group compared with the feladilimab–pembrolizumab group for those with low baseline ctDNA [low ctDNA, HR: 2.26 (95% CI, 1.09, 4.7); high ctDNA, HR: 0.68 (95% CI, 0.33, 1.4); Fig. 3B]. TMB and other baseline biomarker characteristics evaluated did not associate with treatment response. Molecular responders (>50% ctDNA reduction against baseline) were found in both the feladilimab–pembrolizumab and placebo–pembrolizumab groups [54.8% and 41.5%, respectively, when removing NE samples from each arm (feladilimab–pembrolizumab, NE = 6; placebo–pembrolizumab, NE = 5)]; in both groups, molecular response was positively associated with PFS [HR: 0.36 (95% CI, 0.10–1.25); HR: 0.37 (95% CI, 0.13–1.02), respectively, and RECIST ORR, Fig. 3C and D].

Previously reported germline-based genetic biomarkers of immunotherapy response were balanced between treatment arms (Supplementary Table S5) but failed to demonstrate predictive value (Supplementary Figs. S1B and S2A). In the BEP1 population, 134 samples with sufficient tumor tissue were analyzed. The tumor inflammation gene signature (TIS) was balanced between treatment arms and within ctDNA high/low subgroups, with a trend toward improved outcomes in the TIS-high subgroup in both arms (Supplementary Fig. S3A). Tumor microenvironment (TME) features, including several immune cell gene signatures, were balanced between treatment arms and within ctDNA high/low subgroups in each arm (Supplementary Fig. S3B). No additional TME factors or tumor mutational profiles were identified that would explain the differential response between treatment arms (Supplementary Fig. S3C).

## Discussion

INDUCE-3 aimed to determine if the addition of feladilimab to pembrolizumab 1L treatment could improve efficacy in patients with PD-L1-positive R/M HNSCC. Following an unblinded safety and efficacy data review from an interim analysis, the IDMC recommended stopping accrual as the prespecified protocol-defined statistical futility was met, with no evidence of a favorable treatment response with feladilimab–pembrolizumab across primary or key secondary endpoints. The rationale for combining treatments included the antitumor activity of feladilimab in conjunction with PD-1 blockade demonstrated in nonclinical models (13, 22). A promising signal was reported in the HNSCC expansion cohort of the INDUCE-1 study for feladilimab monotherapy in PD-1/PD-L1–experienced HNSCC and in combination with pembrolizumab in previously treated PD-1/PD-L1–naïve HNSCC (13, 23).

Counterintuitively, in INDUCE-3, a more rapid decline in PFS and OS was apparent with feladilimab–pembrolizumab than with placebo–pembrolizumab. For OS, separation between treatment arms was more noticeable in the PD-L1 CPS ≥ 20 subgroup. Data were immature at the analysis, with a high degree of early censoring across both arms in event-driven analyses, and the IDMC believed that extending the study was unlikely to change the outcomes observed in the interim analysis. Overall, these findings suggest that patients responded better to pembrolizumab alone than in combination with feladilimab. HRQoL data were consistent with efficacy findings.

*In vitro* expression of PD-L1 and ICOS-L is inversely correlated within conventional dendritic cells infiltrating HNSCC tumors, with high expression being mutually exclusive (24). Furthermore, ICOS can be expressed on both effector and regulatory T cells in head and neck cancer. Stimulation of regulatory T cells by feladilimab may outweigh any beneficial actions on effector cells; based on RNA sequencing data, regulatory T cells, CD8^+^ T cells, and other immune cell populations were balanced between treatment arms at baseline, and no on-treatment samples were available to determine changes after treatment.

Interestingly, high baseline ctDNA trends with improved responses to feladilimab–pembrolizumab (after adjustment for PD-L1 and HPV status) were observed. Results suggest a possible differential effect of treatment arms based on baseline ctDNA levels, in which immune agonism in a high ctDNA disease-burdened patient population may have benefit; however, the limited sample size impacts firm conclusions. The overall results may have been partially influenced by the imbalance of activity based on ctDNA status of the feladilimab–pembrolizumab combination in the low-ctDNA subgroup. However, these exploratory analyses in a subset of patients with available samples must be interpreted with caution, as the biological explanation remains unclear. The observed difference warrants a separation of baseline ctDNA high and low subgroups in future HNSCC studies. Molecular responders demonstrated improved outcomes with both treatments, consistent with previous reports on immunotherapy outcomes (25). Despite previous studies suggesting a baseline TMB association with immunotherapy outcomes in patients with HNSCC (26, 27), this was not observed in this study. Similarly, previously reported germline biomarkers (*HLA*) variation, skin autoimmunity PRSs, as well as TME factors were not found to be predictive of response to immunotherapy in HNSCC.

Some efficacy parameters for the placebo–pembrolizumab comparator arm in INDUCE-3 were numerically higher than those observed in the pembrolizumab comparator arm in KEYNOTE-048, suggesting differences between trial populations. For example, in the PD-L1 CPS ≥ 20 subgroup, the ORR was 33.3% for the comparator arm in INDUCE-3, but it was 23% in KEYNOTE-048, suggesting that clinicians are increasingly adept at selecting appropriate patients for single-agent anti–PD-1 therapy (1).

The incidence of AEs and serious AEs was comparable between treatment groups. Notably, a higher proportion of patients had TRAEs in the placebo–pembrolizumab group than in the feladilimab–pembrolizumab group. The median number of treatment cycles was similar between treatment groups, suggesting that AE differences may not be attributed to differences in treatment exposure. However, as per the protocol and prespecified statistical analysis plan, an exposure-adjusted analysis was not performed.

These results raise important doubts about ICOS as a target in HNSCC. Furthermore, this is a cautionary reminder about the complexity of the cancer immunity cycle, the difficulty in selecting anti–PD-1 combinations, and that laboratory and single-arm trials of immunotherapy combinations may not produce accurate efficacy results. Recently, there have been several phase III trials of immunotherapy combinations in HNSCC that also failed to meet their primary endpoint, similar to INDUCE-3. Pembrolizumab plus epacadostat treatment (KEYNOTE-669/ECHO-304) resulted in a similar ORR to pembrolizumab monotherapy and SOC. Study enrollment was discontinued early due to findings from KEYNOTE-252; as such, sample sizes were small, and no biomarker analyses were performed (28). Nivolumab plus ipilimumab (CheckMate 651) failed to meet its primary endpoint of OS, with no statistical improvement in OS versus SOC in all randomly assigned or CPS ≥ 20 populations (6). Similarly, durvalumab plus tremelimumab in the 1L (KESTREL) and 2L (EAGLE) HNSCC settings did not result in any statistical improvements in OS versus durvalumab monotherapy or SOC (29, 30). These findings highlight the potential limitations of moving into phase III studies based on single-arm data. Randomized phase II trials such as INDUCE-3, with an adaptive study design, are critical for determining efficacy before proceeding to phase III, with the potential to save considerable time and resources by identifying treatments unlikely to show significant benefit at an earlier stage in development. Additionally, identifying biomarkers associated with response outcomes at an earlier stage would be beneficial ahead of progressing to phase III.

In summary, this study demonstrated no evidence of a treatment effect in favor of feladilimab–pembrolizumab across all primary and key secondary efficacy endpoints in the mITT and PD-L1 ≥ 20 populations. Based on these results, no further clinical investigation of feladilimab in HNSCC is planned.

## Supplementary Material

Supplementary Figure S1 Baseline measurements of A). TMB and B). ctDNA

Supplementary Figure S2 HLA pharmacogenetic analyses in patients treated with pembrolizumab + placebo (mITT population; post-hoc analyses)

Supplementary Figure S3 Tumor gene expression and genomic profiling (post-hoc analyses)

Supplementary Table S1 Representativeness of study participants

Supplementary Table S2 Summary of TTD in pain and physical function (mITT population and PD-L1 CPS≥20 subgroup)

Supplementary Table S3 Treatment-related AEs by preferred term (≥2% of patients, Safety population)

Supplementary Table S4 Summary of AEs by maximum severity grade (Safety population)

Supplementary Table S5 Comparison of baseline germline genetic biomarkers between treatment arms (post-hoc analyses)

## Data Availability

GSK makes available anonymized individual participant data and associated documents from interventional clinical studies that evaluate medicines upon approval of proposals submitted to https://www.gsk-studyregister.com/en/. To access data for other types of GSK-sponsored research, for study documents without patient-level data, and for clinical studies not listed, please submit an inquiry via the website.
