# Supplementary material for: INDUCE-3: A Randomized Phase II/III Study of First-line Feladilimab plus Pembrolizumab in Patients with Recurrent/Metastatic Head and Neck Squamous Cell Carcinoma
Source: Clin Cancer Res. 2025 Dec 22;32(6):1087–99. doi: 10.1158/1078-0432.CCR-25-1197 (PMC13012248; doi:10.1158/1078-0432.CCR-25-1197)
Supplement: Supplementary Figure S1 — Baseline measurements of A). TMB and B). ctDNA [file ccr-25-1197_supplementary_figure_s1_suppfs1.docx]

**Supplementary Figure 1. Baseline measurements of A). TMB and B). ctDNA**


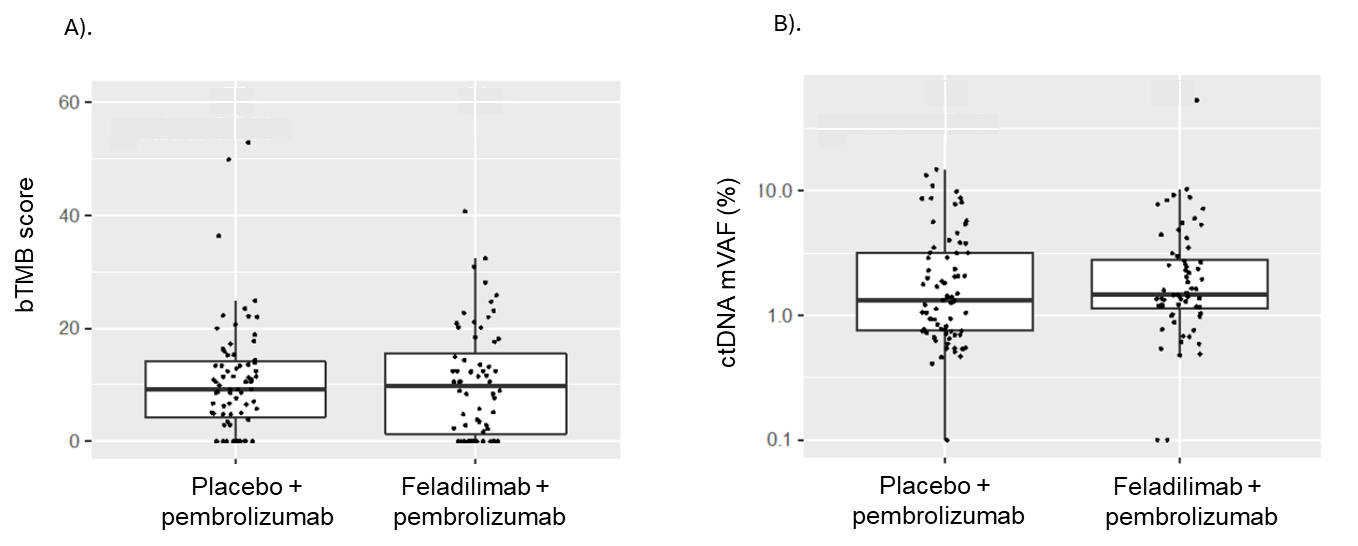


bTMB, baseline tumor mutational burden; ctDNA, circulating tumor DNA; mVAF, mean variant allele frequency
