# Supplementary material for: INDUCE-3: A Randomized Phase II/III Study of First-line Feladilimab plus Pembrolizumab in Patients with Recurrent/Metastatic Head and Neck Squamous Cell Carcinoma
Source: Clin Cancer Res. 2025 Dec 22;32(6):1087–99. doi: 10.1158/1078-0432.CCR-25-1197 (PMC13012248; doi:10.1158/1078-0432.CCR-25-1197)
Supplement: Supplementary Figure S2 — HLA pharmacogenetic analyses in patients treated with pembrolizumab + placebo (mITT population; post-hoc analyses) [file ccr-25-1197_supplementary_figure_s2_suppfs2.docx]

**Supplementary Figure 2. HLA pharmacogenetic analyses in patients treated with pembrolizumab + placebo (mITT population; post-hoc analyses)**

**A.**
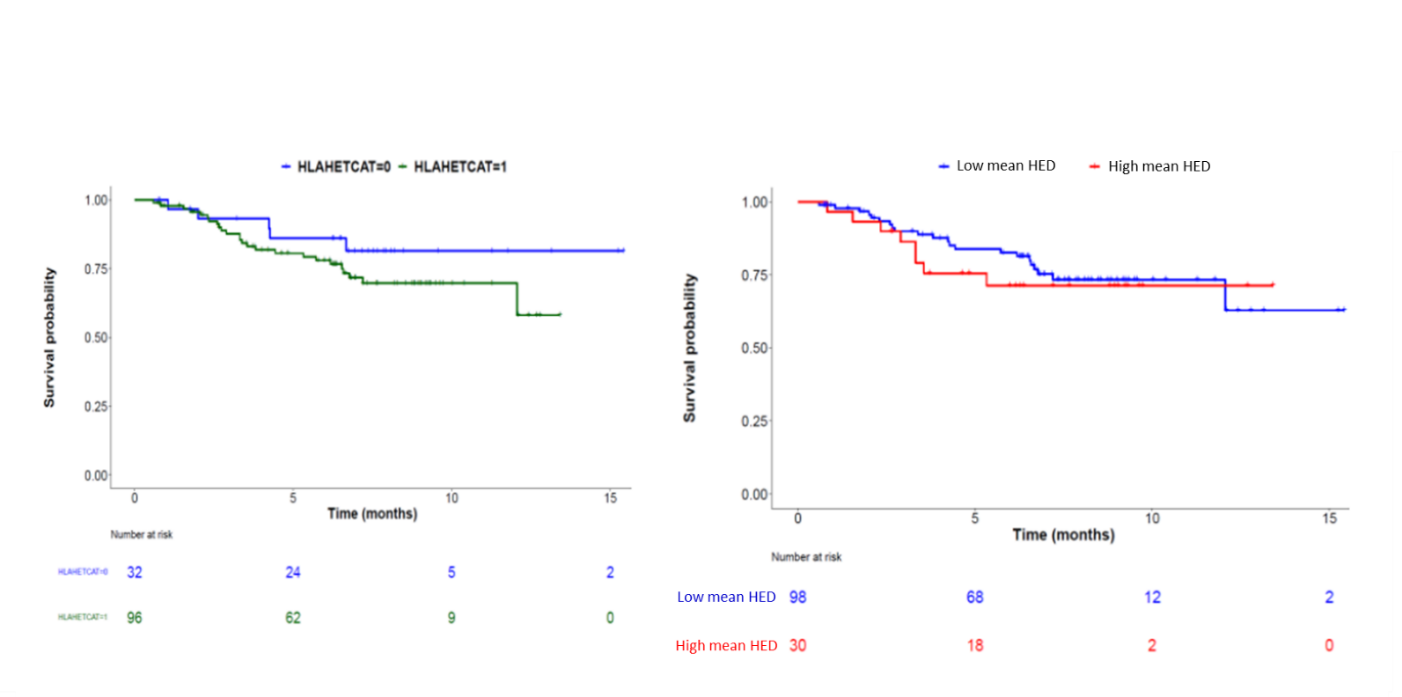


**B.**


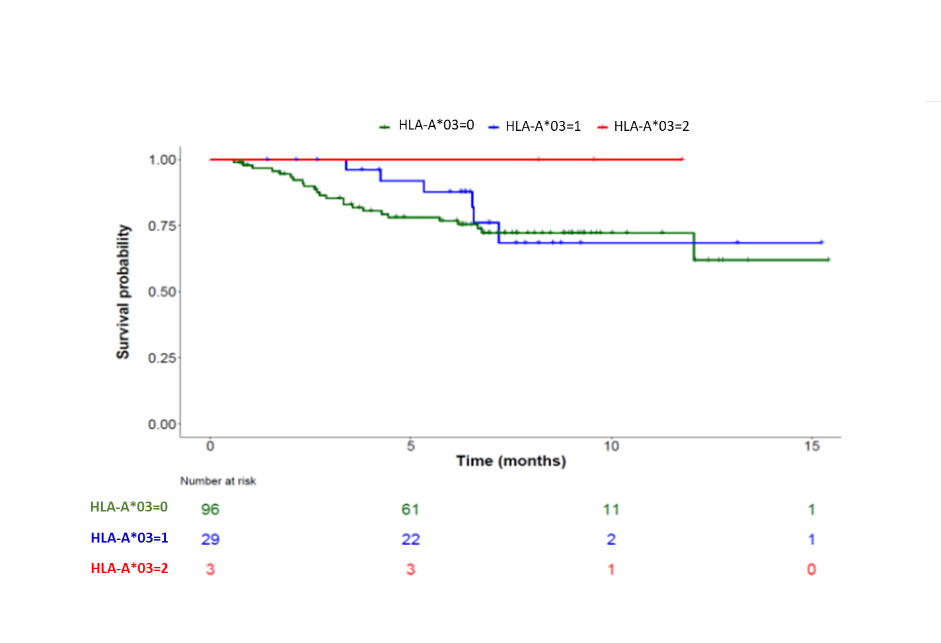


(A) OS Kaplan-Meier curves for HLA heterozygosity and HED. (B) OS Kaplan-Meier curves for HLA-A*03 status. HLA heterozygosity was defined as heterozygous in all of HLA-A, B, and C loci, versus homozygous in at least one of HLA-A, B, or C. Participants were defined as ‘high’ when mean HED was greater than or equal to the top quartile, and ‘low’ was defined as mean HED less than the top quartile. For HLA-A*03 status, participants were stratified according to the number of HLA-A*03 alleles. HED, HLA evolutionary divergence; HLA, human leukocyte antigen; mITT, modified intention-to-treat; OS, overall survival.
