# Supplementary material for: INDUCE-3: A Randomized Phase II/III Study of First-line Feladilimab plus Pembrolizumab in Patients with Recurrent/Metastatic Head and Neck Squamous Cell Carcinoma
Source: Clin Cancer Res. 2025 Dec 22;32(6):1087–99. doi: 10.1158/1078-0432.CCR-25-1197 (PMC13012248; doi:10.1158/1078-0432.CCR-25-1197)
Supplement: Supplementary Figure S3 — Tumor gene expression and genomic profiling (post-hoc analyses) [file ccr-25-1197_supplementary_figure_s3_suppfs3.docx]

**Supplementary Figure 3. Tumor gene expression and genomic profiling (post-hoc analyses)**

**
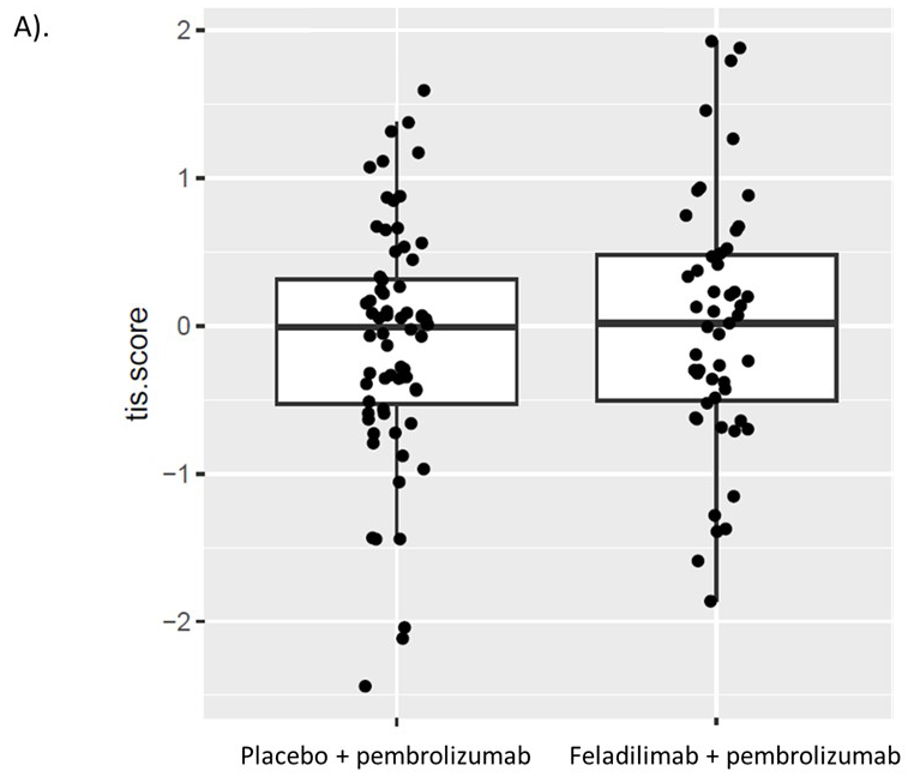
**

**
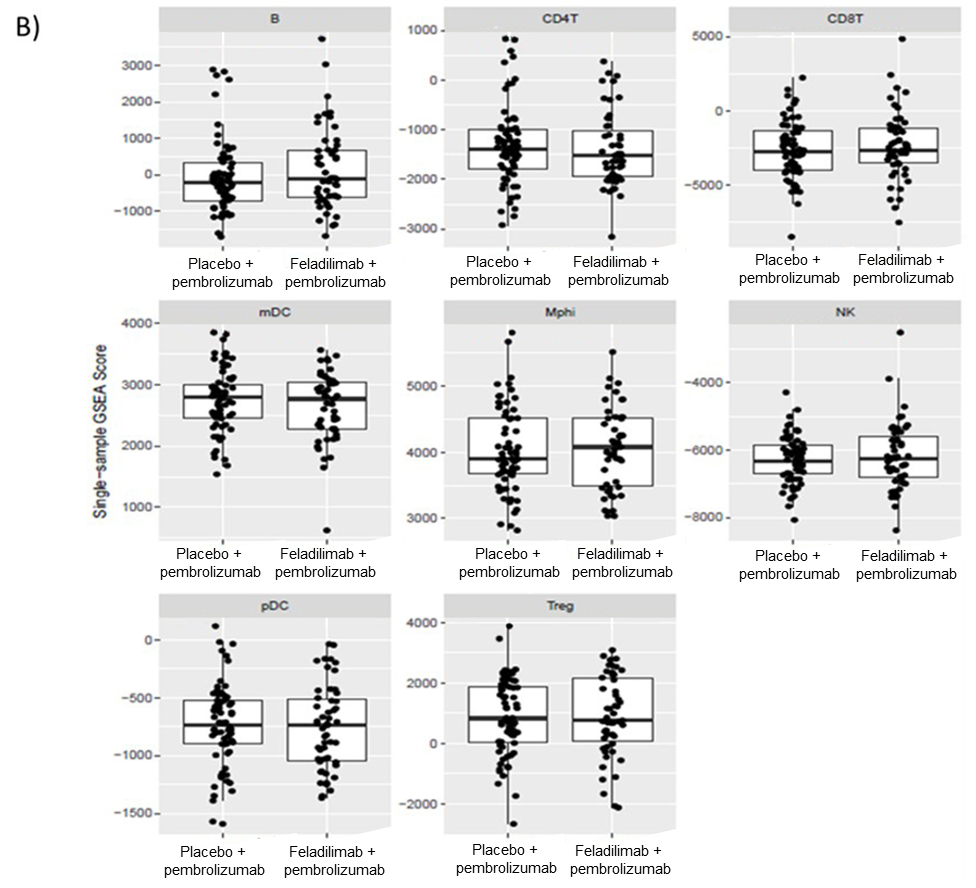
**

**
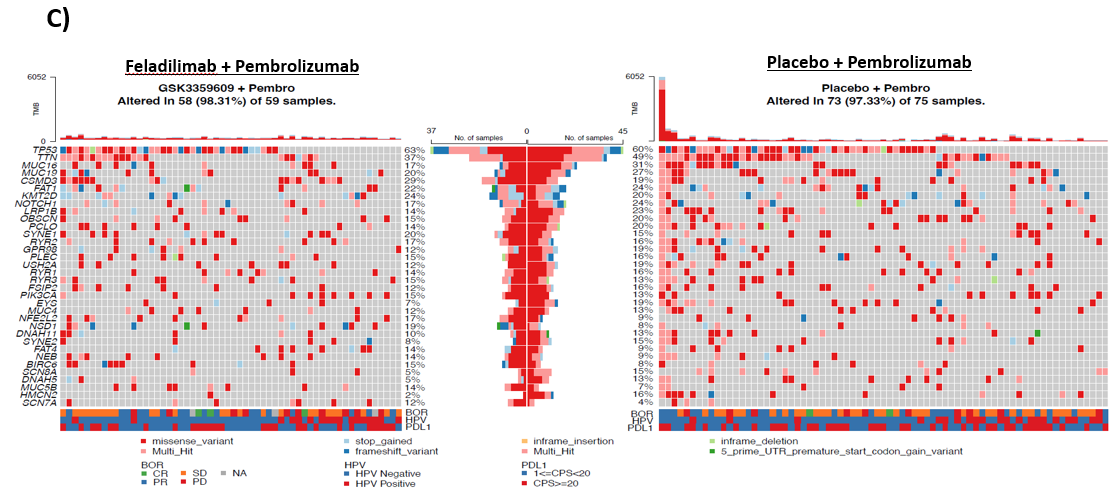
**

(A) Gene expression analysis by RNAseq. TIS score from baseline samples with comparable mean values between treatment arms. (B) GSEA score from RNAseq data evaluating mean immune cell populations between treatment arms. (C) DNA sequencing analysis from baseline tumor samples identifying the most frequent tumor genetic alterations between treatment arms overlaid on BOR, HPV status, and PD-L1 status.

BOR, best overall response; CD4/8, cluster of differentiation 4/8; CPS, combined positive score; CR, complete response; GSEA, gene set enrichment analysis; HPV, human papillomavirus; mDC, monocyte dendritic cells; Mphi, macrophages; NA, not applicable; NK, natural killer cells; PD, progressive disease; pDC, plasmacytoid dendritic cells; PD-L1, programmed death-ligand 1; PR, partial response; SD, stable disease; TIS, tumor inflammation signature; Treg, T-cell regulatory cells.
