# Supplementary material for: INDUCE-3: A Randomized Phase II/III Study of First-line Feladilimab plus Pembrolizumab in Patients with Recurrent/Metastatic Head and Neck Squamous Cell Carcinoma
Source: Clin Cancer Res. 2025 Dec 22;32(6):1087–99. doi: 10.1158/1078-0432.CCR-25-1197 (PMC13012248; doi:10.1158/1078-0432.CCR-25-1197)
Supplement: Supplementary Table S1 — Representativeness of study participants [file ccr-25-1197_supplementary_table_s1_suppts1.docx]

**Supplementary Table 1. Representativeness of study participants**

| **Cancer type(s)/ subtype(s)/ stage(s)/ condition** | Recurrent/metastatic head and neck squamous cell carcinoma |
| --- | --- |
| **Conditions related to:** | |
| **Sex** | Globally, HNSCC is more common in men than women, with a male-to-female ratio of approximately 3:1. HPV-positive HNSCC is even higher in men, with a male-to-female ratio of approximately 3–6:1. |
| **Age** | The median age at the time of diagnosis is 64, with approximately 50% of patients diagnosed between the ages of 55 and 74. |
| **Race/Ethnicity** | In the USA between 2018–2021, 66.3% of HNSCC cases were in White patients; however, overall survival was significantly higher in White compared with Black patients. |
| **Geography** | According to WHO 2022 data, HNSCC is the 6^th^ most common cancer globally. The highest incidence rates for the HNSCC population representative of those enrolled in this trial are in Asia (61.1%), followed by Europe (19.0%), and North America (8.3%). |
| **Other considerations** | Tobacco and alcohol consumption are risk factors for HNSCC, accounting for approximately 70% of cases. HPV is estimated to cause approximately 60–70% of oral pharyngeal cancers in the USA. |
| **Overall representativeness in this study** | Overall, the population in our study (81% male, median age 63.0 years, 57% HPV-positive oropharynx cancer, 81% current/former smokers) is mostly representative of the HNSCC population. Consistently with global incidence, patients enrolled were primarily White (75%), and therefore results are less representative of other ethnic groups. This was a global study with patients enrolled primarily in Europe (53%) and only approximately 20% of patients enrolled from Asia, the continent with the highest reported incidence rates globally. |
