# Supplementary material for: INDUCE-3: A Randomized Phase II/III Study of First-line Feladilimab plus Pembrolizumab in Patients with Recurrent/Metastatic Head and Neck Squamous Cell Carcinoma
Source: Clin Cancer Res. 2025 Dec 22;32(6):1087–99. doi: 10.1158/1078-0432.CCR-25-1197 (PMC13012248; doi:10.1158/1078-0432.CCR-25-1197)
Supplement: Supplementary Table S2 — Summary of TTD in pain and physical function (mITT population and PD-L1 CPS≥20 subgroup) [file ccr-25-1197_supplementary_table_s2_suppts2.docx]

**Supplementary Table 2. Summary of TTD in pain and physical function (mITT population and PD-L1 CPS≥20 subgroup)**

|  | **mITT population** | | **PD-L1 CPS≥20 subgroup** | |
| --- | --- | --- | --- | --- |
|  | **Feladilimab plus pembrolizumab (n=157)** | **Placebo plus pembrolizumab (n=156)** | **Feladilimab plus pembrolizumab (n=70)** | **Placebo plus pembrolizumab (n=69)** |
| **TTD in pain: number of patients, n (%)** | | | | |
| Deterioration | 47 (30) | 47 (30) | 14 (20) | 20 (29) |
| Censored | 110 (70) | 109 (70) | 56 (80) | 49 (71) |
| **Estimates for TTD in pain, months^a^** | | | | |
| Median  (95% CI) | 6.3 (5.1–N/A) | 10.4 (6.3–N/A) | N/A (5.1–N/A) | 12.0 (6.3–N/A) |
| Adjusted HR^b^ estimate  (95% CI) | 1.17 (0.78–1.77) | _ | 1.00  (0.50–2.00) | _ |
| **TTD in physical function: number of patients, n (%)** | | | | |
| Deterioration | 47 (30) | 64 (41) | 18 (26) | 29 (42) |
| Censored | 110 (70) | 92 (59) | 52 (74) | 40 (58) |
| **Estimates for TTD in physical function (months)^a^** | | | | |
| Median  (95% CI) | 4.9  (3.5–7.7) | 4.9  (3.0–6.3) | 4.9  (2.1–N/A) | 4.9  (3.1–N/A) |
| Adjusted HR^b^ estimate  (95% CI) | 0.91  (0.62–1.34) | _ | 1.09  (0.60–2.00) | _ |

Data cutoff April 27, 2021. TTD in pain assessed by EORTC IL51 pain domain and TTD in physical function assessed by PROMIS PF-8c. mITT population included all randomized patients who received the study intervention whether or not randomized but excluded those who were first dosed or randomized after the date of requesting immediate discontinuation of feladilimab and placebo. ^a^CI estimated using the Brookmeyer-Crowley method; ^b^An HR <1 indicates a lower risk with feladilimab plus pembrolizumab compared with placebo plus pembrolizumab; CI, confidence interval; CPS, combined positive score; EORTC IL, European Organisation for Research and Treatment of Cancer Item Library; HR, hazard ratio; mITT, modified intention-to-treat; N/A, not applicable; PD-L1 CPS, programmed cell death ligand-1 combined positive score; PROMIS PF-8c, PRO Measurement Information System Physical function-8c; TTD, time to deterioration.
