# Supplementary material for: INDUCE-3: A Randomized Phase II/III Study of First-line Feladilimab plus Pembrolizumab in Patients with Recurrent/Metastatic Head and Neck Squamous Cell Carcinoma
Source: Clin Cancer Res. 2025 Dec 22;32(6):1087–99. doi: 10.1158/1078-0432.CCR-25-1197 (PMC13012248; doi:10.1158/1078-0432.CCR-25-1197)
Supplement: Supplementary Table S3 — Treatment-related AEs by preferred term (≥2% of patients, Safety population) [file ccr-25-1197_supplementary_table_s3_suppts3.docx]

**Supplementary Table 3. Treatment-related AEs by preferred term (≥2% of patients, Safety population)**

| **n (%)** | **Feladilimab plus pembrolizumab (n=159)** | **Placebo plus pembrolizumab (n=156)** | **Total  (n=315)** |
| --- | --- | --- | --- |
| **Any event** | **76 (48)** | **89 (57)** | **165 (52)** |
| Fatigue | 12 (8) | 16 (10) | 28 (9) |
| Rash | 7 (4) | 16 (10) | 23 (7) |
| Decreased appetite | 8 (5) | 14 (9) | 22 (7) |
| Hypothyroidism | 10 (6) | 12 (8) | 22 (7) |
| Pruritus | 6 (4) | 11 (7) | 17 (5) |
| AST increased | 7 (4) | 6 (4) | 13 (4) |
| Nausea | 6 (4) | 7 (4) | 13 (4) |
| ALT increased | 6 (4) | 6 (4) | 12 (4) |
| Asthenia | 6 (4) | 6 (4) | 12 (4) |
| Diarrhea | 6 (4) | 5 (3) | 11 (3) |
| Weight decreased | 5 (3) | 6 (4) | 11 (3) |
| Headache | 5 (3) | 4 (3) | 9 (3) |
| Arthralgia | 4 (3) | 4 (3) | 8 (3) |
| Anemia | 6 (4) | 1 (<1) | 7 (2) |
| Hyperthyroidism | 2 (1) | 5 (3) | 7 (2) |
| Blood ALP increased | 4 (3) | 2 (1) | 6 (2) |
| Dyspnea | 5 (3) | 1 (<1) | 6 (2) |
| Blood TSH increased | 1 (<1) | 4 (3) | 5 (2) |
| Constipation | 1 (<1) | 4 (3) | 5 (2) |
| Hypokalemia | 4 (3) | 1 (<1) | 5 (2) |
| Pneumonitis | 2 (1) | 3 (2) | 5 (2) |
| Stomatitis | 2 (1) | 3 (2) | 5 (2) |
| Vomiting | 2 (1) | 3 (2) | 5 (2) |

Data cutoff April 27, 2021. Safety population includes all patients who received at least one dose of allocated study treatment. AE, adverse event; ALP, alkaline phosphatase; ALT, alanine aminotransferase; AST, aspartate aminotransferase; TSH, thyroid stimulating hormone.
