# Supplementary material for: INDUCE-3: A Randomized Phase II/III Study of First-line Feladilimab plus Pembrolizumab in Patients with Recurrent/Metastatic Head and Neck Squamous Cell Carcinoma
Source: Clin Cancer Res. 2025 Dec 22;32(6):1087–99. doi: 10.1158/1078-0432.CCR-25-1197 (PMC13012248; doi:10.1158/1078-0432.CCR-25-1197)
Supplement: Supplementary Table S4 — Summary of AEs by maximum severity grade (Safety population) [file ccr-25-1197_supplementary_table_s4_suppts4.docx]

**Supplementary Table 4. Summary of AEs by maximum severity grade (Safety population)**

| **n (%)** | **Feladilimab plus pembrolizumab (n=159)** | **Placebo plus pembrolizumab (n=156)** | **Total  (n=315)** |
| --- | --- | --- | --- |
| **Maximum grade (any event)** | | | |
| Grade 1 | 38 (24) | 27 (17) | 65 (21) |
| Grade 2 | 47 (30) | 51 (33) | 98 (31) |
| Grade 3 | 40 (25) | 43 (28) | 83 (26) |
| Grade 4 | 5 (3) | 4 (3) | 9 (3) |
| Grade 5 | 15 (9) | 15 (10) | 30 (10) |
| Grade ≥3 | 60 (38) | 62 (40) | 122 (39) |
| **Grade ≥3 AEs (≥3% in either treatment group)** | | | |
| Dysphagia | 5 (3) | 7 (4) | 12 (4) |
| Tumor hemorrhage | 5 (3) | 6 (4) | 11 (3) |
| Hypercalcemia | 4 (3) | 5 (3) | 9 (3) |
| Anemia | 3 (2) | 6 (4) | 9 (3) |
| Pneumonia | 6 (4) | 2 (1) | 8 (3) |
| Dyspnea | 5 (3) | 3 (2) | 8 (3) |
| Decreased appetite | 1 (<1) | 4 (3) | 5 (2) |

Data cutoff April 27, 2021. Safety population includes all patients who received at least one dose of allocated study treatment. AE, adverse event.
