# Supplementary material for: INDUCE-3: A Randomized Phase II/III Study of First-line Feladilimab plus Pembrolizumab in Patients with Recurrent/Metastatic Head and Neck Squamous Cell Carcinoma
Source: Clin Cancer Res. 2025 Dec 22;32(6):1087–99. doi: 10.1158/1078-0432.CCR-25-1197 (PMC13012248; doi:10.1158/1078-0432.CCR-25-1197)
Supplement: Supplementary Table S5 — Comparison of baseline germline genetic biomarkers between treatment arms (post-hoc analyses) [file ccr-25-1197_supplementary_table_s5_suppts5.docx]

**Supplementary Table 5. Comparison of baseline germline genetic biomarkers between treatment arms (post-hoc analyses)**

| **Biomarker** | **Placebo plus pembrolizumab** | **Feladilimab plus pembrolizumab** |
| --- | --- | --- |
| HLA Heterozygosity, Yes (%) | 96 (75%) | 83 (74%) |
| HED Mean Score, mean (SD) | 6.32 (2.23) | 6.39 (2.13) |
| HLA-A*03 allele count (0/1/2) | 96/29/3 | 89/20/3 |
| PRS AD [p<1e-8], mean (SD) | -0.00771 (0.01677) | -0.00645 (0.01654) |
| PRS psoriasis [p<0.001], mean (SD) | -0.01132 (0.00534) | -0.01174 (0.00511) |
| PRS psoriasis [p<0.01], mean (SD) | -0.00361 (0.00203) | -0.00369 (0.00266) |
| PRS psoriasis [p<0.1], mean (SD) | -0.00037 (0.00092) | -0.00020 (0.00129) |
| PRS vitiligo [p<1e-8], mean (SD) | -0.00049 (0.01255) | 0.00174 (0.01339) |
| PRS vitiligo [p<1e-7], mean (SD) | 0.00012 (0.01058) | 0.00194 (0.01188) |
| PRS vitiligo [p<1e-5], mean (SD) | 0.00293 (0.00522) | 0.00399 (0.00579) |

AD, Alzheimer’s disease; HLA, human leukocyte antigens; HED, HLA evolutionary divergence; PRS, polygenic risk scores; SD, standard deviation.
